# Supplementary material for: vsgseq2: an updated pipeline for analysis of the diversity and abundance of population-wide Trypanosoma brucei VSG expression
Source: Wellcome Open Res. 2025 Dec 12;10:672. [Version 1] doi: 10.12688/wellcomeopenres.24932.1 (PMC12756598; doi:10.12688/wellcomeopenres.24932.1)
Supplement: Peer review history

# Reviewer report: Reviewer response for version 1

**DOI:** 10.21956/wellcomeopenres.27463.r142461

**Published:** 2026-01-08

**Authors:** TERRY K SMITH

**Affiliations:** 1 University of St Andrews, St Andrews, Scotland, UK

The authors present an update version of VSGseq  (2) for the analysis of VSG transcripts of the *T.brucei* parasites. The new pipeline improves accuracy and  reproducibility, while making it more user friendly.

 The 3 way comparison  of the benchmark  and in vivo data, helps to validate vsgseq2, which showed more clarity when identify the mosaic forms of VSGs than vsgseq.

 Due to the modified pipeline , the run time of vsgseq2 is significantly reduce and therefore is greener as less power is required.

 Can the authors please add a section in the methods about the statistical analysis they did and in the legends of the appropriate figures state what "**" versus "***" means.

Are the conclusions about the tool and its performance adequately supported by the findings presented in the article?

Yes

Is the rationale for developing the new software tool clearly explained?

Yes

Is the description of the software tool technically sound?

Partly

Are sufficient details of the code, methods and analysis (if applicable) provided to allow replication of the software development and its use by others?

Yes

Is sufficient information provided to allow interpretation of the expected output datasets and any results generated using the tool?

Yes

Reviewer Expertise:

Molecular and Biochemical Parasitology 30 years working with T. brucei

I confirm that I have read this submission and believe that I have an appropriate level of expertise to confirm that it is of an acceptable scientific standard.

# Reviewer report: Reviewer response for version 1

**DOI:** 10.21956/wellcomeopenres.27463.r142463

**Published:** 2026-01-06

**Authors:** Francesca Florini

**Affiliations:** 1 Department of Microbiology and Immunology, Weill Cornell Medical College, New York, New York, USA

*Trypanosoma brucei* is an extracellular parasite and, as such, is continuously exposed to the host immune system. To evade immune recognition, parasites are coated with variant surface glycoproteins (VSGs) and can switch between different VSG variants, enabling the establishment of chronic infections. Each parasite expresses only a single VSG at any given time, while different parasites within the same host express different VSGs, generating extensive antigenic diversity.

 To disentangle VSG diversity within a host, some of the authors previously developed the VSGSeq method. In this manuscript, they present an improved version of this pipeline, termed vsgseq2. Overall, the method appears to capture VSG expression in parasite populations more accurately than the original approach, and the manuscript addresses an important technical challenge in the field. I have a few comments and points that would benefit from clarification.

- In the Methods, the authors state that vsgseq2 supports both single-end and paired-end sequencing. However, it is unclear which approach was actually used for the data presented in this manuscript. Were the vsgseq2 libraries sequenced as single-end or paired-end? Have the authors compared the performance of the two approaches using this pipeline?
- Why is the number of VSGs detected by the VSGSeq_combined approach so high? While I understand the loss of reads due to multimapping, it is less clear why a substantial fraction of reads would be assigned to incorrect VSGs.  
- My understanding is that VSGSeq_Individual also discards multimapped reads, yet it still performs relatively well in terms of mapping reliability. This suggests that the limitations of VSGSeq_combined may extend beyond multimapping alone. This interpretation is further supported by the mouse infection experiments shown in Figure 3, where VSGSeq_Individual and vsgseq2 show good agreement. The authors may want to discuss more explicitly what the specific advantages of vsgseq2 are compared to VSGSeq_Individual.
- I am somewhat skeptical of allowing multimapped reads to be assigned to the most highly expressed gene when dealing with multigene families, as this could potentially lead to misinterpretation, particularly in more complex populations than those shown in Figure 2. That said, as the authors mention in the Discussion, the pipeline is customizable, and this problem could presumably be addressed depending on experimental requirements.
- In Figure 2D, some VSGs (3/11/417) appear to lose substantially more reads than the other VSGs. Do the author know whether these VSGs are more similar to other VSGs at the sequence level, potentially explaining this behavior/
- Figure 5 is a bit unclear. I think it would benefit from a clearer and more descriptive legend. The current legend explains the data but does not adequately describe the figure itself, making it difficult to follow without extensive reference to the main text.
- Although beyond the scope of this study, it would be interesting to comment on the potential of combining vsgseq2 with long-read sequencing approaches to directly resolve ambiguous reads and highly similar VSGs.

Are the conclusions about the tool and its performance adequately supported by the findings presented in the article?

Yes

Is the rationale for developing the new software tool clearly explained?

Yes

Is the description of the software tool technically sound?

Yes

Are sufficient details of the code, methods and analysis (if applicable) provided to allow replication of the software development and its use by others?

Yes

Is sufficient information provided to allow interpretation of the expected output datasets and any results generated using the tool?

Partly

Reviewer Expertise:

Parasitology, Antigenic Variation

I confirm that I have read this submission and believe that I have an appropriate level of expertise to confirm that it is of an acceptable scientific standard.

# Reviewer report: Reviewer response for version 1

**DOI:** 10.21956/wellcomeopenres.27463.r142464

**Published:** 2026-01-06

**Authors:** Mark Carrington

**Affiliations:** 1 Biochemistry, University of Cambridge, Cambridge, UK

*Trypanosoma brucei *can maintain long term infections of mammals through antigenic variation of the VSG, an immunodominant cell surface protein. Individual cells express one VSG but a population can be a mixture of cells expressing different VSGs. Analysis VSG expression in a population has been achieved by selective RT-PCR of VSG mRNAs and then illumina sequencing of the mixture. The paper describes an updated workflow for the identification and quantitation of VSG mRNAs from such a mixture.

 The paper is sound, but I have a few suggestions:

 1. Figure 3B. This is an analysis of a test mixture that was a tenfold dilution series of clones expressing different VSGs.  As such, it would be better if the y-axis was on a log10 scale, this would make visualisation of minor components accessible.

 2. Figure 4b.  On my back of the envelope calculation, the CO2 saving (~1000 mg) is equivalent to 30 average human exhalations (30 mg / breathing cycle). Perhaps the authors could alter the y-axis to use this as a unit as no context for the amount of CO2 saved is given.

 Methods: 'sequenced in triplicate' biological or technical repeats?

 Methods:  were the spliced leader and 14-mer sequences removed from reads before any assembly?

 Methods: 'Transcripts below 0.01% of the population were removed' Please clarify as some cells in library A were present at 0.0001%. Were the reads from these removed? Did this step remove minor VSGs in the mouse analysis?

 Minor comments

 Abstract:  'VSG encoding genes account for ~10% of the T. brucei genome' If this is intact VSGs later on ~400 is mentioned, this would be 5%. If it includes VSG pseudogenes it is more than 10%.

 Abstract: 'almost infinite' It is either infinite, or it isn't, it can't be almost. 

Are the conclusions about the tool and its performance adequately supported by the findings presented in the article?

Yes

Is the rationale for developing the new software tool clearly explained?

Yes

Is the description of the software tool technically sound?

Yes

Are sufficient details of the code, methods and analysis (if applicable) provided to allow replication of the software development and its use by others?

Yes

Is sufficient information provided to allow interpretation of the expected output datasets and any results generated using the tool?

Yes

Reviewer Expertise:

NA

I confirm that I have read this submission and believe that I have an appropriate level of expertise to confirm that it is of an acceptable scientific standard, however I have significant reservations, as outlined above.

# Reviewer report: Reviewer response for version 1

**DOI:** 10.21956/wellcomeopenres.27463.r142465

**Published:** 2025-12-31

**Authors:** Sara Silva Pereira

**Affiliations:** 1 Universidade Católica Portuguesa, Oeiras, Portugal

*Trypanosoma brucei* evades host immunity by switching among a vast repertoire of variant antigens, the VSGs. Due to their large genomic repertoires and impressive levels of recombination, T. brucei VSG analysis from conventional sequencing data is barely possible. In 2015, VSGSeq revolutionized the field by providing a way of obtaining expressed VSG repertoires. Now, the original authors and collaborators have improved the analysis pipeline that makes VSGSeq possible. The authors present **vsgseq2**, a Nextflow-based workflow that improves accuracy, efficiency, and quantification of VSG expression. In this paper, benchmarking and in vivo analyses are shown to provide evidence that vsgseq2 better reconstructs VSG repertoires with lower computational cost.

 I installed the software and ran it on the test data using Conda and Docker as described in the README file without problems. I did not test the software with additional data or with alternative containers. My comments are exclusively based on my experience.

 Below I provide some minor comments that, in my opinion, could make the work stronger and easier to understand.

 Abstract:

*“Trypanosoma brucei is an extracellular eukaryotic parasite that causes sleeping sickness in humans and Nagana, Surra and Dourine in livestock, game animals and horses.” *I suggest writing nagana, surra and dourine in lower case.

 Methods:

1. Control libraries A and B were sequenced by VSGseq, correct? I suggest to clearly mention it in the text.
2. For control library A, Tb427VSG-417 was included at a 0.0001% of a population of 1x10^6 parasites. This equals to 1 parasite in total. The error associated with such a dilution can be quite considerable. How did the authors deal with this challenge?
3. “ *To account for issues with the VSGseq pipeline when it was run in parallel across multiple samples…” *what issues? I suggest to clearly describe some of the issues encountered to provide context.
4. Please clarify what is “ *cd-hit-est run” *and its purpose for the uninitiated reader.
5. *“manually annotated as false positives”: *Although the authors reference the original VSGseq paper (2015), it would be helpful to have some examples of false positives included in the text. I would also suggest informing the number of original and new false positive sequences.
6. “ *The result of these changes is that vsgseq2 chooses a champion sequence based on transcript abundance, rather than sequence length.” *What happens if the champion is different between samples but due to small variations in TPM in the original transcripts that make up the cluster?
7. *“…ultimately meaning more VSG sequences will be clustered and represented by a single sequence in the final VSGome” *How does the pipeline differ between truly distinct sequences (ie. mosaics) and multiple versions of the same VSG arising from multi-mapped reads?
8. Figure1: In vsgseq2, it is unclear when the analyses progresses directly from “Trim reads” to “Quantify with Salmon”, and also what is the decision process behind going from “Concatenate VSGs” to either “Quantify with Salmon” or “Cluster with cd-hit-est”. I suggest adding a bit more information and potential questions/answers, like in vsgseq-combined.

Results:

1. In panels A and B of Figure 2, there are stars overlapping the “A” and “B” box labels. I suggest slightly moving them down.
2. In both panels of Figure 4, the stars are not aligned with the two bars and are missing the square bracket that was common to previous figures. For the sake of consistency, I suggest editing it.
3. In figures 4 and 5, the panel labels are not capitalized like in previous figures. This is also the case in the boxes of figure 5E, when Fig. 5B-D are mentioned). For the sake of consistency, I suggest editing it.
4. In figure 5B-E the font is too small. In panels b-d, the font appears different from the remaining panels/figures. For the sake of consistency, I suggest editing it.

Software:

1. I suggest to add instructions on how to install wget under “Download test data” in the README file: (e.g. conda install anaconda::wget)
2. I think that Trinity 2.15.1 does not exist for osx-arm64 on conda (Apple Silicon), so users should use the docker alternative. I suggest this to be clearly mentioned in the README file.

Are the conclusions about the tool and its performance adequately supported by the findings presented in the article?

Yes

Is the rationale for developing the new software tool clearly explained?

Yes

Is the description of the software tool technically sound?

Yes

Are sufficient details of the code, methods and analysis (if applicable) provided to allow replication of the software development and its use by others?

Partly

Is sufficient information provided to allow interpretation of the expected output datasets and any results generated using the tool?

Partly

Reviewer Expertise:

Trypanosome antigenic diversity, Trypanosome infection biology.

I confirm that I have read this submission and believe that I have an appropriate level of expertise to confirm that it is of an acceptable scientific standard.

# Reviewer report: Reviewer response for version 1

**DOI:** 10.21956/wellcomeopenres.27463.r142469

**Published:** 2025-12-30

**Authors:** Bibo Li

**Affiliations:** 1 Cleveland State University, Cleveland, Ohio, USA

*Trypanosoma brucei* is a kinetoplastid parasite that infects humans, livestock, game animals and horses. It undergoes antigenic variation to evade the mammalian host’s immune response, and a key process of antigenic variation is to express immunologically distinct VSG surface coat. In this manuscript, Oldrieve et al. revised the pipeline for analyzing HTS data to examine VSG diversity profiles of *T. brucei* populations. The original VSGseq analyses (VSGseq-combined and VSGseq-individual) were compared to the new VSGseq2 analysis. Control *T. brucei* populations with known fractions of parasites that express different VSGs were examined and VSG count numbers, percent of mapped reads and quantification results were shown to validate the improved VSGseq2 approach. In addition, previous HTS data from longitudinal chronic infections were also analyzed using the three pipelines, and the results were compared. In general, VSGseq2 gives much improved results than VSGseq, particularly VSGseq-combined. VSGseq2 identifies the highly expressed VSG more accurately and gives better quantification results among the *T. brucei* population. In addition, VSGseq2 uses Salmon, the latest version of Trinity, and newer versions of Python and Nextflow. VSGseq2 also has an improved calculation efficiency.

 This reviewer only has a couple of minor concerns:

1. VSGseq2 is more conservative in analyzing assembled donor and mosaic VSGs when compared to VSGseq. However, the advantage or disadvantage of this feature needs to be explained more clearly. It would be more useful if the authors give specific instructions on how to adjust the parameters to make VSGseq2 more or less conservative.
2. In the title, “Trypanoosoma brucei” should be in the italic font.

Are the conclusions about the tool and its performance adequately supported by the findings presented in the article?

Yes

Is the rationale for developing the new software tool clearly explained?

Yes

Is the description of the software tool technically sound?

Yes

Are sufficient details of the code, methods and analysis (if applicable) provided to allow replication of the software development and its use by others?

Yes

Is sufficient information provided to allow interpretation of the expected output datasets and any results generated using the tool?

Yes

Reviewer Expertise:

parasitology, molecular biology, chromosome biology

I confirm that I have read this submission and believe that I have an appropriate level of expertise to confirm that it is of an acceptable scientific standard.
